# Immunotherapy related endocrinopathy of the pineal and pituitary gland that resolved following discontinuation of treatment: case report

**DOI:** 10.1007/s00234-025-03687-x

**Published:** 2025-07-08

**Authors:** Anish Bhandari, Ilaria Vittoria De Martini, Samuel N. Rogers, Aparna Nallagangula, Hasan Ozgur

**Affiliations:** 1 Banner - University Medical Center Tucson, Tucson, AZ United States; 2 University of Arizona, Tucson, AZ United States

**Keywords:** Immune checkpoint inhibitors, Immune-related adverse events, Pineal gland, Hypophysitis, Pituitary gland

## Abstract

Immune checkpoint inhibitors (ICI) have been integrated into various tumor treatment protocols, including melanoma. Endocrinopathies related to ICI have been well-documented, with most common sites of involvement being thyroid, pituitary, adrenal, and pancreas. We report a 65-year-old female with metastatic melanoma who developed endocrinopathy of the pineal gland and pituitary gland following treatment with ICI. Although metastatic disease was considered in the differential diagnosis, the MRI findings in conjunction with resolution upon discontinuation of immunotherapy was most consistent with inflammatory etiology. A comprehensive literature search yielded no reports of immunotherapy-induced endocrinopathy of the pineal gland, suggesting that this may be potentially the first reported case. Understanding the clinical and imaging findings of immune-related adverse events in patients undergoing immunotherapy is crucial to ensure proper diagnosis and subsequent treatment plans.

##  Introduction

Immune checkpoint inhibitors (ICIs) enhance the immune system’s ability to eliminate tumor cells by blocking inhibitory checkpoints that suppress T-cell activity. ICIs may lead to excessive immune responses, known as immune-related adverse events (irAEs) [1]. Endocrinopathies, including pituitary inflammation (hypophysitis), are a recognized subset of irAEs. ICIs can cause enlargement of the gland and stalk due to inflammation and immune-mediated damage [2]. Ipilimumab, a Cytotoxic T-Lymphocyte Antigen-4 (CTLA-4) inhibitor, and nivolumab, a Programmed Cell Death Protein 1 (PD-1) inhibitor, are commonly combined to produce robust anti-tumor responses. Combination therapy has a higher incidence and severity of hypophysitis, typically manifesting within weeks to months after therapy initiation [3, 4]. Symptoms include headache, visual disturbances, and hormonal imbalances such as adrenal insufficiency, hypothyroidism, and hypogonadism.

MRI is the preferred modality for evaluating pituitary abnormalities in patients receiving ICI therapy. MRI findings of ICI-related hypophysitis include pituitary enlargement, stalk thickening, homogenous or heterogenous gadolinium enhancement, T2 hypointensity, and absence of normal posterior pituitary T1 hyperintensity (“bright spot”) [2, 5]. These imaging features, in conjunction with clinical and laboratory findings, are crucial for differentiating hypophysitis from other pathologies, particularly metastatic disease. Although pineal gland involvement in ICI therapy did not appear in our literature search, we expect similar inflammatory changes such as enlargement and hyperenhancement.

## Case presentation

**Fig. 1:**
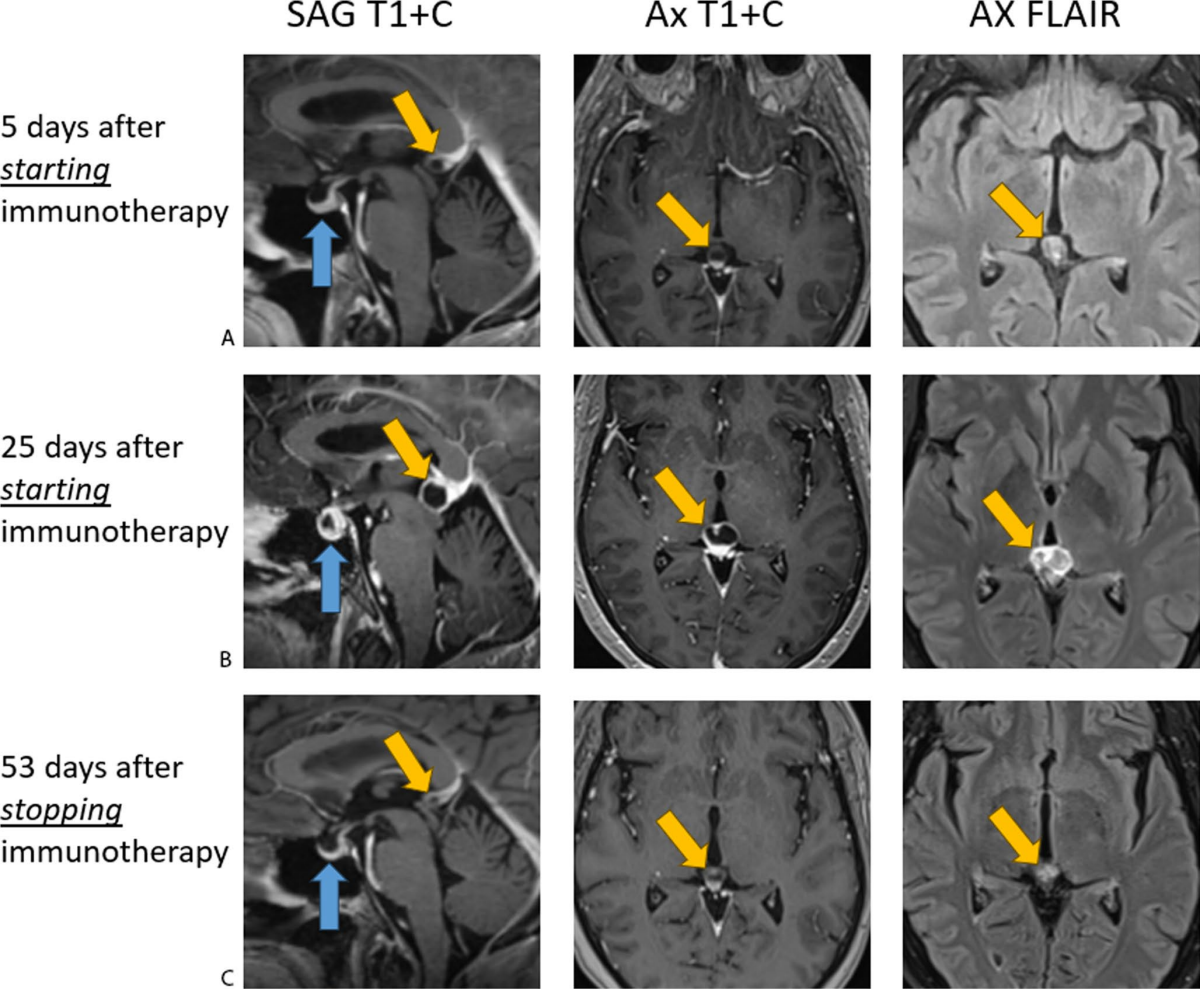
Demonstrates the Sagittal T1 post contrast, Axial T1 post contrast, and axial FLAIR sequences of the brain. **A** is the baseline demonstrating two tiny, non-enhancing simple pineal cysts with normal pituitary gland. **B** shows interval increase in size of the pituitary gland and pineal cysts. The pituitary gland was enhancing heterogeneously. There is also increased peripheral enhancement of the pineal cysts. **C** is 53 days after stopping immunotherapy demonstrating complete resolution of the pituitary and pineal abnormalities

A 65-year-old female presented to the emergency department with chest pain. Initial CT thorax revealed a left infrahilar mass. Subsequent PET-CT imaging identified bilateral lung masses and hilar lymphadenopathy. Biopsy confirmed metastatic melanoma. Two weeks later, the patient began a combination ICI therapy of Ipilimumab (3 mg/kg) and Nivolumab (1 mg/kg). Staging contrast-enhanced brain MRI showed an enhancing 4 mm right frontal lobe lesion, concerning for metastatic disease. An incidental finding was two tiny, non-enhancing simple pineal cysts (Fig. 1a) with normal pituitary gland.

Three weeks after immunotherapy initiation, the patient returned to the Emergency Department with new-onset headaches and blurry vision. Brain MRI revealed significant enlargement of both the pineal and the pituitary glands (Fig. 1b). The pineal gland demonstrated peripheral enhancement of the cysts, and the pituitary gland showed heterogeneous enhancement. The frontal lobe lesion slightly increased in size. Lumbar puncture (LP) showed elevated protein levels (130 mg/mL; normal range 15–60 mg/mL), concerning for aseptic meningitis. Additional irAEs included severe colitis and mild skin rash. The patient did not have any clinical symptoms related to pineal dysfunction. Immunotherapy was discontinued, and the patient was treated with high-dose prednisone starting with 100 mg and then tapered weekly to 30 mg. Due to worsening diarrhea, steroid therapy was discontinued. Prednisone was later resumed at 80 mg prednisone daily and then tapered to 70 mg after a week with improved tolerance.

Follow-up MRI three months from presentation showed resolution of pituitary abnormalities and significant reduction in pineal cyst size after steroid treatment, supporting an inflammatory rather than metastatic etiology (Fig. 1c). Transition was made to Trametinib, a mitogen-activated protein kinase (MEK) inhibitor, alongside the prednisone taper. Despite clinical improvement and no additional lesions seen on MRI, treatment was discontinued due to a decrease in ejection fraction from 73 to 57%. Approximately two months later, numerous new intracranial lesions developed. The pineal and pituitary glands remained unremarkable. Stereotactic radiation therapy for the frontal lobe lesions was performed, and two months later imaging showed progression of intracranial disease with no pineal or pituitary changes. The patient was then transitioned to hospice care.

## Discussion

IrAEs affects up to 40% of patients and may involve the thyroid, pituitary gland, adrenal gland and pancreas, with symptoms ranging from mild to life threating [4]. Endocrinopathies often present with vague and nonspecific symptoms, making early diagnosis difficult yet crucial for patient prognosis and quality of life. IrAEs in non-endocrine organs may resolve over time; however, in endocrine organs they often lead to permanent hormone deficiencies, requiring lifelong replacement therapy.

Ipilimumab and nivolumab are monoclonal antibodies that enhance T-cell activity on tumor cells by acting on CTLA-4 and PD-1 receptors [6]. This immune activation may also provoke autoimmune responses targeting normal tissues. Hypophysitis may be partially mediated by type 2 hypersensitivity reactions, as supported in animal models showing CTLA-4 receptor expression in mice pituitary glands [7]. 

The pineal gland is an endocrine organ that produces melanin, which plays an essential role in circadian rhythm. Pineal cysts are common incidental findings on MRI. Most pineal cysts remain stable in size, although there are reports describing a small number of incidental pineal cysts increasing in size [8, 9]. Al-Holou et al. reported the only predictive factor for growth of a pineal cyst being a younger age of the patients. No correlation was seen between growth and cyst morphology or signal characteristics. Some proposed mechanisms for pineal cyst growth are coalescence of smaller cysts, hemorrhage into a cyst, and hormonal influences. Two reports of enlarging pineal cysts becoming symptomatic during pregnancy were made, with hormonal and menstrual cycle changes being noted as potential causes [10, 11]. Another two reports of idiopathic pinealitis resulted in obstructive hydrocephalus in patients not on ICIs [12, 13]. Chronic inflammatory pinealitis was diagnosed by histopathology and there were no other diseases found to explain the inflammatory response.

In our patient, pituitary findings were compatible with immunotherapy related hypophysitis and pinealitis, and metastatic disease was highly unlikely. This is because the symptoms started three weeks after initiation of immunotherapy and completely resolved following discontinuation. The patient’s headaches and blurry vision, although non-specific, were likely related hypophysitis as there were no other abnormalities in the MRI study to explain them. There were no symptoms to suggest pineal dysfunction. There has been reports of pineal cyst enlargement due to hormonal changes, such as in pregnancy [10]. In our patient, the enlargement of the pineal cyst may be related to hormonal changes related to pituitary dysfunction or the direct effect of immunotherapy agents similar to other endocrine organs. The main limitation of our report lack of biopsy or autopsy confirmation.

## Conclusion

Immune checkpoint inhibitors can cause potentially life-threatening irAEs, including neurologic disorders and endocrinopathies. This case highlights the importance of differentiating between metastatic disease and immunotherapy-related inflammatory changes. MRI pattern of immunotherapy related hypophysitis is well described and we favored this diagnosis over metastatic disease. While MRI patterns of ICI-related hypophysitis have been described, pineal gland changes have not been reported in this context. Pinealitis may be considered to be added to the list of ICI related endocrinopathies. Careful monitoring and a high index of suspicion are crucial in providing an accurate diagnosis of inflammatory changes versus neoplastic process.

## Data Availability

No datasets were generated or analysed during the current study.
